# Overview of osteoimmunology: What's happened? And what's going on?

**DOI:** 10.1186/ar3567

**Published:** 2012-02-09

**Authors:** Hiroshi Takayanagi

**Affiliations:** 1 Tokyo Medical and Dental University, ERATO, Japan

## 

The interaction between the immune and skeletal systems has long been acknowledged, but molecular mechanisms linking the two systems have not been demonstrated until recently. Investigation into autoimmune arthritis as well as the various bone phenotypes found in mice deficient in immunomodulatory molecules has highlighted the importance of the dynamic interplay between the two systems and brought about a rapid evolution of the field of osteoimmunology [1]. In bone loss in autoimmune arthritis, IL-17-producing helper T (T_H_17) cells play a major role by inducing RANKL [2]. Maintenance and mobilization of hematopoietic cells are regulated by bone cells. In addition to cellular interactions via cytokines, the immune and skeletal systems share various molecules, including transcription factors, signaling molecules and membrane receptors. RANKL stimulates osteoclastogenesis through NFATc1 in cooperation with immunoglobulin-like receptors. Here I will discuss emerging topics in osteoimmunology including the mechanisms underlying bone cell communication: osteocyte RANKL [3] and inhibition of bone formation by osteoclast Sema4D [4].
